# Preoperative plasma D-dimer independently predicts survival in patients with pancreatic ductal adenocarcinoma undergoing radical resection

**DOI:** 10.1186/s12957-021-02281-8

**Published:** 2021-06-09

**Authors:** Haoda Chen, Fanlu Li, Siyi Zou, Junjie Xie, Jun Zhang, Xiaxing Deng, Hao Chen, Baiyong Shen

**Affiliations:** Department of General Surgery, Pancreatic Disease Center, Ruijin Hospital, Shanghai Jiao Tong University School of Medicine, 197 Ruijin Er Road, Shanghai, 200025 China

**Keywords:** D-dimer, Pancreatic ductal adenocarcinoma (PDAC), Pancreatic surgery, Prognostic factors

## Abstract

**Background:**

Elevated plasma D-dimer levels have been reported as an unfavorable prognostic indicator in many solid tumors. However, there are limited relevant studies in pancreatic cancer patients following radical surgery, and the clinical significance remains controversial. The aim of this study was to investigate the clinical and prognostic significance of preoperative plasma D-dimer in patients with pancreatic ductal adenocarcinoma (PDAC) undergoing resection.

**Methods:**

A retrospective analysis was performed on all patients who consecutively underwent radical surgery for PDAC by laparotomy or robotic surgery from December 2011 to December 2018. Baseline clinicopathologic characteristics, preoperative laboratory parameters, and follow-up information were collected. Univariate and multivariate analyses were performed to analyze the prognostic value of preoperative plasma D-dimer.

**Results:**

Among 1351 patients, elevated preoperative plasma D-dimer levels (≥ 0.55 ng/mL) were found in 417 (30.9%) patients. Three hundred twelve (23.09%) underwent minimally invasive robotic pancreatectomy. The median overall survival (OS) of patients with elevated D-dimer levels was 6.3 months shorter than that of patients with normal D-dimer levels (15.0 months vs 21.3 months, p < 0.001). Multivariate analysis showed that elevated D-dimer levels independently predicted poorer OS (hazard ratio, 1.33; 95% confidence interval, 1.17-1.51, p < 0.001). Subgroup analysis demonstrated that D-dimer was a reliable prognostic factor in patients who underwent R0 resection. In addition, integration of D-dimer, carbohydrate antigen 19-9 (CA19-9), and NLR provided a better prognostic model for PDAC patients before operation.

**Conclusion:**

An elevated preoperative plasma D-dimer level was a reliable independent prognostic factor for OS in patients with PDAC undergoing resection. Combination of D-dimer, CA19-9, and NLR can enhance the prognostic accuracy before operation.

**Supplementary Information:**

The online version contains supplementary material available at 10.1186/s12957-021-02281-8.

## Introduction

Pancreatic cancer, a highly aggressive malignancy, is the fourth leading cause of cancer-related deaths in developed countries, with an overall 5-year survival rate of 9% [1]. Radical surgical resection remains the only treatment for long-term survival, but tumor recurrence occurs in almost 80% of patients [2]. In addition, patients with the same pathological staging and therapeutic procedure show differential prognosis after radical resection [3, 4]. Although Ca19-9 has been widely used as an adverse prognostic biomarker in pancreatic cancer, approximately 6 to 22% of the population have no or low secretion of CA19-9 [5, 6]. The heterogeneity of prognostic outcomes highlights the urgent need for a more robust biomarker for long-term oncological outcomes, which could help identify high-risk patients, decide individual therapeutic strategies, and facilitate close patient follow-up [4, 7–10].

Since Trousseau first described the relationship between cancer and thromboembolic disease in 1865, activation of the coagulation system via host-tumor interactions has been found in almost all types of cancer [11–13]. In addition, the tumor-induced hypercoagulation state is not simply an epiphenomenon but is also intrinsically involved in enhanced tumor growth, angiogenesis, metastasis, and the systemic inflammatory response [11, 12, 14]. D-dimer is a soluble degradation product that results from the systematic degeneration of vascular thrombi by the fibrinolytic system [15]. The presence of D-dimer molecules is suggestive of intravascular coagulation since it can only be generated after thrombin formation and subsequent breakdown of cross-linked fibrin [16]. Therefore, plasma D-dimer levels are used routinely to monitor the risk of deep venous thrombosis (DVT), pulmonary embolism (PE), aortic dissection, and as an aid in the diagnosing disseminated intravascular coagulation (DIC) [15]. Recently, elevated plasma D-dimer levels have been reported to represent an unfavorable prognostic indicator in lung cancer, colorectal cancer, pancreatic cancer, and several other solid tumors [17]. Preoperative high level of D-dimer is also associated with occult hepatic metastases in patients with pancreatic head cancer [18].

However, there are limited relevant studies in pancreatic ductal adenocarcinoma (PDAC) patients following radical surgery, and no studies regarding the prognostic value of preoperative plasma D-dimer in PDAC patients treated with minimally invasive robotic surgery [19, 20]. Therefore, the purpose of this study is to assess the predictive value of D-dimer on postoperative overall survival in patients with PDAC in a high-volume pancreatic center.

## Methods

### Patients

A retrospective cohort analysis was performed on all patients who consecutively underwent radical surgery for PDAC by laparotomy or robotic surgery from December 2011 to December 2018 at the Department of General Surgery, Shanghai Ruijin Hospital. The inclusion and exclusion criteria were as follows: (1) pathologically proven PDAC; (2) complete clinicopathologic and follow-up data; (3) no evidence of distant metastasis or macroscopic residual tumors; (4) no preoperative antitumor treatment; (5) no history of other malignancies; (6) no acute inflammatory diseases; and (7) no anticoagulant therapy before the operation. This study was approved by the review board of Shanghai Ruijin Hospital (No. 2020-361). Informed consent was waived according the committee’s regulations. Data has been reported in line with STROCSS 2019 criteria.

### Data collection

Data were obtained from a prospectively maintained database and electronic medical records in the hospital. The clinical and demographic variables included age, sex, body mass index (BMI), platelet, albumin, neutrophil lymphocyte ratio (NLR), D-dimer, and serum carbohydrate antigen 19-9 (CA19-9) levels. All laboratory parameters were assayed during routine workups before surgery. Plasma D-dimer levels were measured by immunoturbidimetric assays. The normal reference value for plasma D-dimer and CA19-9 in our institution are below 0.55 ng/mL and 37U/mL, respectively.

The standard operative procedures and management strategies were described in our previous studies [21, 22]. The treatment details include preoperative biliary drainage, surgical approach (open/robotic), and the performance of major vessel resection (portal vein, superior mesenteric vein, celiac trunk, or common hepatic artery). The final pathological diagnosis was also documented according to the TNM staging system of the American Joint Committee on Cancer (AJCC), 8th edition. We also recorded whether patients received any form of adjunct chemotherapy. The overall survival (OS) was calculated from the date of surgical resection to the date of death or the last follow-up. The last follow-up time was August 2020.

### Statistical methods

Descriptive statistics were described as frequencies and percentages for categorical variables and as medians with interquartile ranges for continuous variables. Normality was examined using the Shapiro-Wilk test. Baseline characteristics were compared using Pearson Chi-squared test and Kruskal-Wallis rank-sum test. Preoperative biomarkers were dichotomized around their median or dichotomized according to their clinical references in the model. Survival curves were plotted according to the Kaplan-Meier method from the time of surgery to the time of death or last follow-up and were compared using the log-rank test. The Cox proportional hazard regression model was used for univariate and multivariate analyses. The significant statistical variables (p < 0.05) in univariate analysis were included into the multivariate analysis to identify the independent prognostic factors for survival. The forest plot was performed to show the outcome of subgroup analysis. Interactions between subgroups were calculated based on an additive model to investigate whether the effect of D-dimer on clinical outcome was modified by other variables. Additionally, the prognostic accuracy and discriminatory ability of each prognostic model were evaluated by the concordance index (C-index) and Akaike information criterion (AIC), respectively. All analyses were performed using Stata (Stata Corporation, version 15, College Station, TX, USA), with p < 0.05 (two-sided) considered statistically significant.

## Results

### Patients characteristics

A total of 1351 patients with PDAC undergoing radical resection were included in this study. The final study population comprised 503 (37.23%) women and 848 (62.77%) men, with a median age of 63 (58-69) years. Overall, 475 (35.16%) patients had stage I disease, 459 (33.97%) had stage II disease, and 417 (30.87%) had stage III disease. Minimally invasive robotic pancreatic surgeries were performed in 312 (23.09%) patients. Symptomatic DVT was observed in only 3 patients during hospitalization. Further information is described in Supplementary Table 1. The median follow-up time was 43.7 months; the median OS for the entire cohort was 19.0 months and 1024 (75.70%) patients died during the follow-up period. The OS rates at 1, 2, and 3 years were 69.9%, 40.8%, and 26.2%, respectively.

Elevated preoperative plasma D-dimer levels were found in 417 (30.9%) patients. The elevated D-dimer levels were not significantly related with increased postoperative bleeding complications when compared to the normal D-dimer levels (4.32% vs 3.75%, p = 0.619). The analysis of the relationship between D-dimer and other clinicopathologic factors is shown in Table 1. Elevated D-dimer levels were significantly associated with a high platelet level (p = 0.002), a low albumin level (p < 0.001), a high NLR level, a high CA19-9 level (p = 0.005), pancreatic head cancer (p = 0.012), poor tumor differentiation (p = 0.014), and less adjuvant chemotherapy (p < 0.002).

**Table 1:** Correlations between D-dimer and clinicopathologic features of patients

	D-dimer < 0.55 ng/mL (n = 934)	D-dimer ≥ 0.55 ng/mL (n = 417)	p
Age	63 (58-69)	64 (58-70)	0.153
Sex (male)	585 (62.63%)	263 (63.07%)	0.878
BMI, kg/m^2^	22.7 (20.8-24.4)	22.7 (20.3-24.5)	0.467
Platelet, ×10^9^	183 (148-225)	192 (153-247)	**0.002**
Albumin, g/L	39 (36-42)	39 (36-41)	0.089
Obstructive jaundice	301 (32.23%)	180 (43.17%)	**< 0.001**
NLR	2.45 (1.87-3.28)	2.71 (2.03-3.76)	**0.001**
CA19-9, U/mL	137.3 (37.3-387.2)	179.2 (49.0-611.4)	**0.005**
ASA score			0.915
1	412 (44.11%)	184 (44.12%)	
2	505 (54.07%)	224 (53.72%)	
3	17 (1.82%)	9 (2.16%)	
Comorbidity			
Hypertension	343 (36.72%)	172 (41.25%)	0.114
Diabetes mellitus	235 (25.16%)	85 (20.38%)	0.056
Cardiac disease	65 (6.96%)	23 (5.52%)	0.321
Tumor location			**0.012**
Head	572 (61.24%)	285 (68.35%)	
Body/tail	362 (38.76%)	132 (31.65%)	
Surgical approach			0.086
Open	706 (75.59%)	333 (79.86%)	
Robotic	228 (24.41%)	84 (20.14%)	
Major vessel resection	136 (14.56%)	55 (13.19%)	0.504
Neural invasion	798 (85.44%)	357 (85.61%)	0.934
R1 resection, ≤ 1 mm	114 (12.21%)	64 (15.35%)	0.115
T stage			0.674^a^
T1	159 (17.02%)	64 (15.35%)	
T2	428 (45.82%)	192 (46.04%)	
T3	120 (12.85%)	66 (15.83%)	
T4	227 (24.30%)	95 (22.78%)	
N stage			0.521^a^
N0	487 (52.14%)	218 (52.28%)	
N1	360 (38.54%)	142 (34.05%)	
N2	87 (9.31%)	57 (13.67%)	
Differentiation			**0.014**^a^
Well	361 (38.65%)	137 (32.85%)	
Moderate	422 (45.18%)	193 (46.28%)	
Poor	151 (16.17%)	87 (20.86%)	
Adjuvant chemotherapy	556 (59.53%)	210 (50.34%)	**0.002**

*BMI*, body mass index; *NLR*, neutrophil lymphocyte ratio; *CA19-9*, serum carbohydrate antigen 19-9

*p* value < 0.05 indicates statistical significance (in bold)

^a^The chi-squared test for trend was used for the comparison of ordinal variables

### Preoperative plasma D-dimer as an independent prognostic factor

**Fig. 1:**
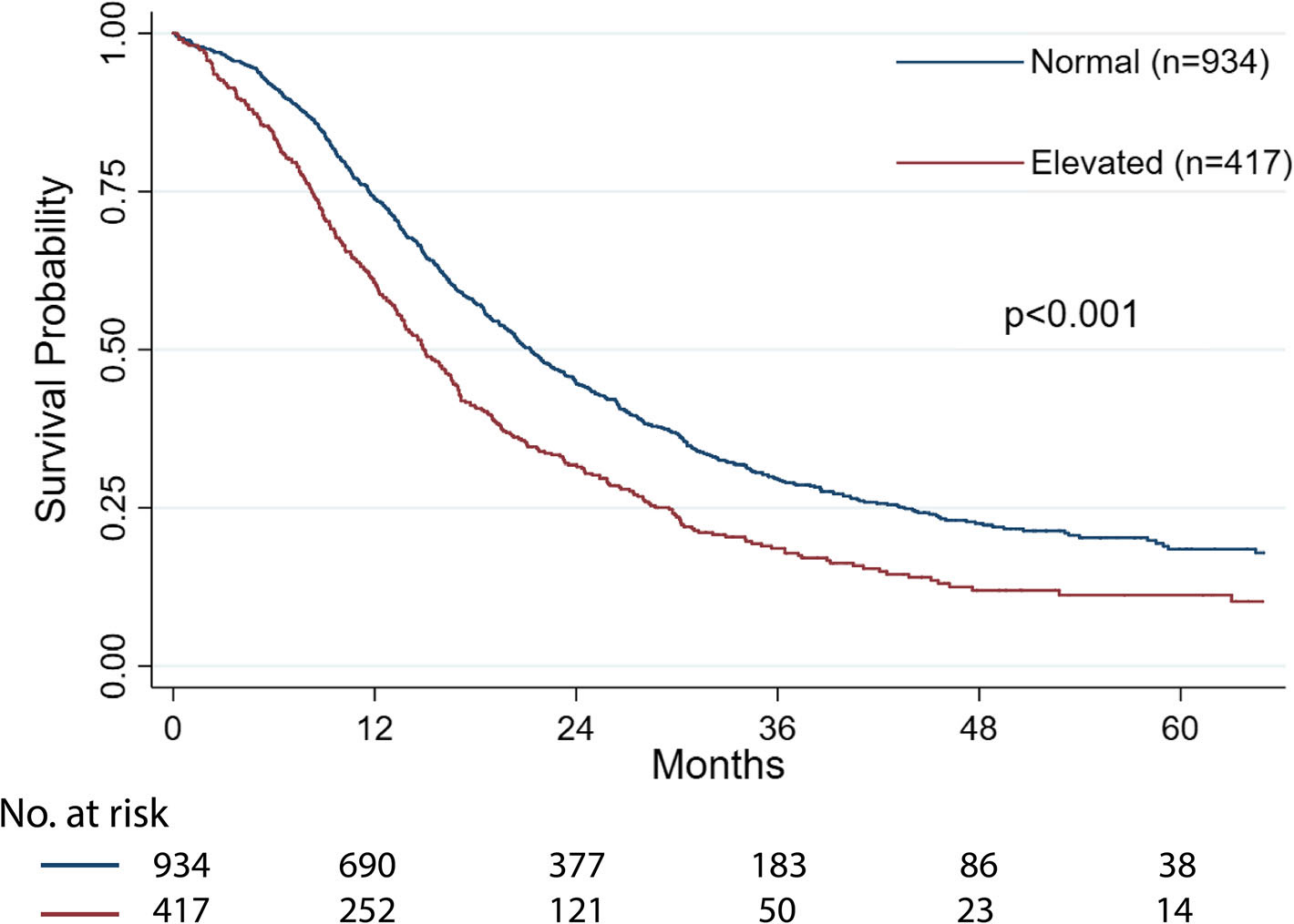
Kaplan-Meier survival curve of overall survival according to preoperative plasma D-dimer level in PDAC patients

In the univariate analysis, elevated D-dimer levels were found to be an adverse prognostic factor for OS (hazard ratio (HR) 1.47, 95% confidence interval (CI) 1.29-1.67, p < 0.001, Table 2). Figure 1 shows the associated Kaplan-Meier curves for D-dimer in relation to overall survival for the whole cohort. The median OS of patients with elevated D-dimer levels was 6.3 months shorter than that of patients with normal D-dimer levels (15.0 months vs 21.3 months, p < 0.001). In the multivariate analysis, preoperative plasma D-dimer was identified as an independent prognostic factor (HR 1.33, 95% CI 1.17-1.51, P < 0.001, Table 2). In addition, a high NLR, a high CA19-9 level, nerve plexus invasion, R1 resection, T3-T4 stage, N1-N2 stage, poor differentiation, and adjuvant therapy were also independent predictive factors for OS.

**Table 2:** Univariate and multivariate Cox proportional-hazard regression analysis for overall survival in PDAC patients undergoing resection

	Patients (%)	Univariable analysis		Multivariable analysis	
		Hazard ratio	p	Hazard ratio	p
Age, ≥ 65	591 (43.75%)	1.23 (1.09-1.38)	**0.001**	1.13 (1.00-1.29)	0.057
Sex (male)	848 (62.77%)	1.05 (0.92-1.19)	0.468		
Platelets, ≥ 200 × 10^9^	561 (41.52%)	1.10 (0.98-1.25)	0.110		
NLR, ≥ 3	470 (34.79%)	1.28 (1.12-1.45)	**< 0.001**	1.19 (1.05-1.36)	**0.009**
Albumin, < 40 g/L	742 (54.92%)	1.19 (1.05-1.34)	**0.007**	1.11 (0.98-1.26)	0.116
Obstructive jaundice	481 (35.60%)	1.16 (1.02-1.31)	**0.025**	0.94 (0.81-1.09)	0.424
D-dimer, ≥ 0.55 ng/mL	417 (30.87%)	1.47 (1.29-1.67)	**< 0.001**	1.33 (1.17-1.51)	**< 0.001**
CA19-9, ≥ 37 U/mL	1036 (76.68%)	1.40 (1.23-1.58)	**< 0.001**	1.25 (1.10-1.41)	**0.001**
Tumor location					
Head	857 (63.43%)	Ref.			
Body/tail	494 (36.57%)	0.97 (0.85-1.10)	0.639		
Major vessel resection	191 (14.14%)	1.57 (1.33-1.86)	**<0.001**	1.08 (0.89-1.32)	0.419
Neural invasion	1155 (85.49%)	1.51 (1.26-1.81)	**< 0.001**	1.39 (1.15-1.67)	**0.001**
R1 resection, ≤ 1 mm	178 (13.18%)	1.51 (1.27-1.79)	**< 0.001**	1.39 (1.16-1.65)	**< 0.001**
T stage					
T1-T2	843 (62.40%)	Ref.		Ref.	
T3-T4	508 (37.60%)	1.57 (1.39-1.78)	**< 0.001**	1.54 (1.34-1.80)	**< 0.001**
N stage					
N0	705 (52.18%)	Ref.		Ref.	
N1-N2	646 (47.82%)	1.58 (1.40-1.79)	**< 0.001**	1.49 (1.31-1.69)	**< 0.001**
Differentiation					
Well-moderate	1113 (82.38%)	Ref.		Ref.	
Poor	238 (17.62%)	1.77 (1.52-2.06)	**< 0.001**	1.73 (1.48-2.02)	**< 0.001**
Adjuvant therapy	751 (55.59%)	0.61 (0.54-0.69)	**< 0.001**	0.61 (0.54-0.70)	**< 0.001**

*PDAC*, pancreatic ductal adenocarcinoma; *NLR*, neutrophil lymphocyte ratio; *CA19-9*, serum carbohydrate antigen 19-9

*p* value < 0.05 indicates statistical significance (in bold)

### Prognostic value of D-dimer in different subgroups

**Fig. 2:**
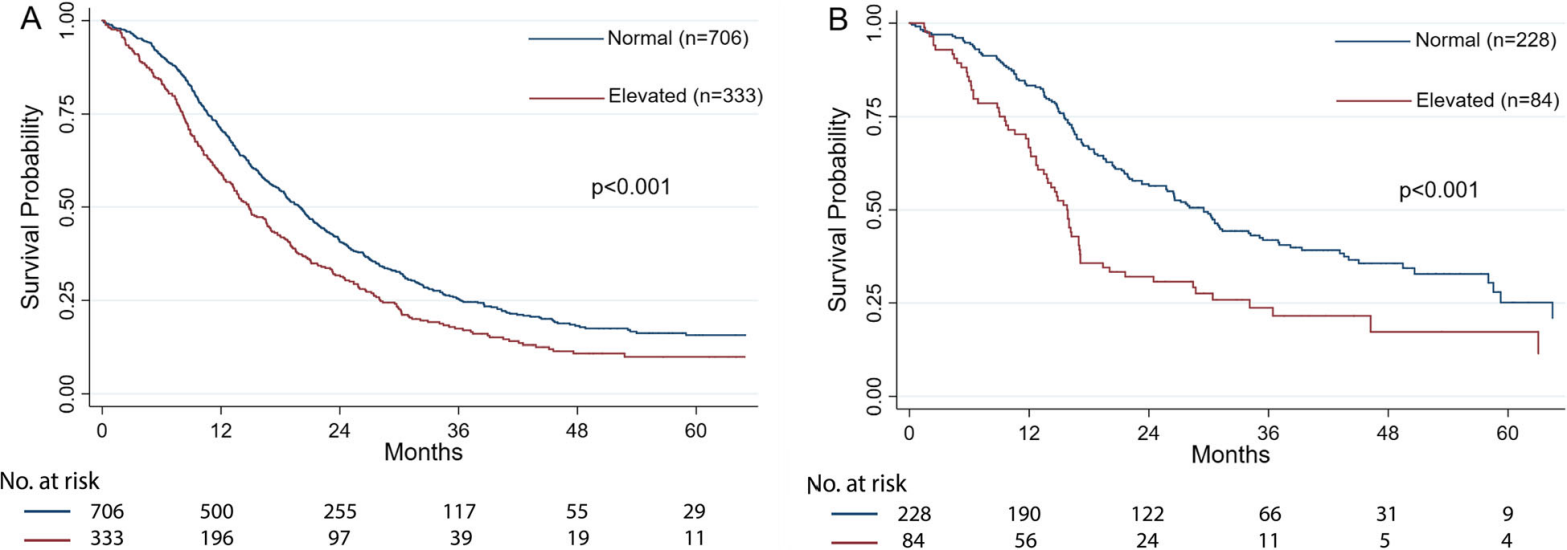
Kaplan-Meier survival curve of overall survival stratified by preoperative serum D-dimer level in the open surgery group (**A**) and the robotic surgery group (**B**)

In the different surgical approach groups, patients with elevated D-dimer levels showed worse OS in both open surgery group and the robotic group (both: p < 0.001; Fig. 2). And D-dimer remained an independent prognostic factor for OS even after adjustment for other prognostic factors (open surgery: HR 1.25, 95% CI 1.09-1.45, p = 0.008; robotic surgery: HR 1.77, 95% CI 1.29-2.44, p < 0001; Supplementary Table 2).

**Fig. 3:**
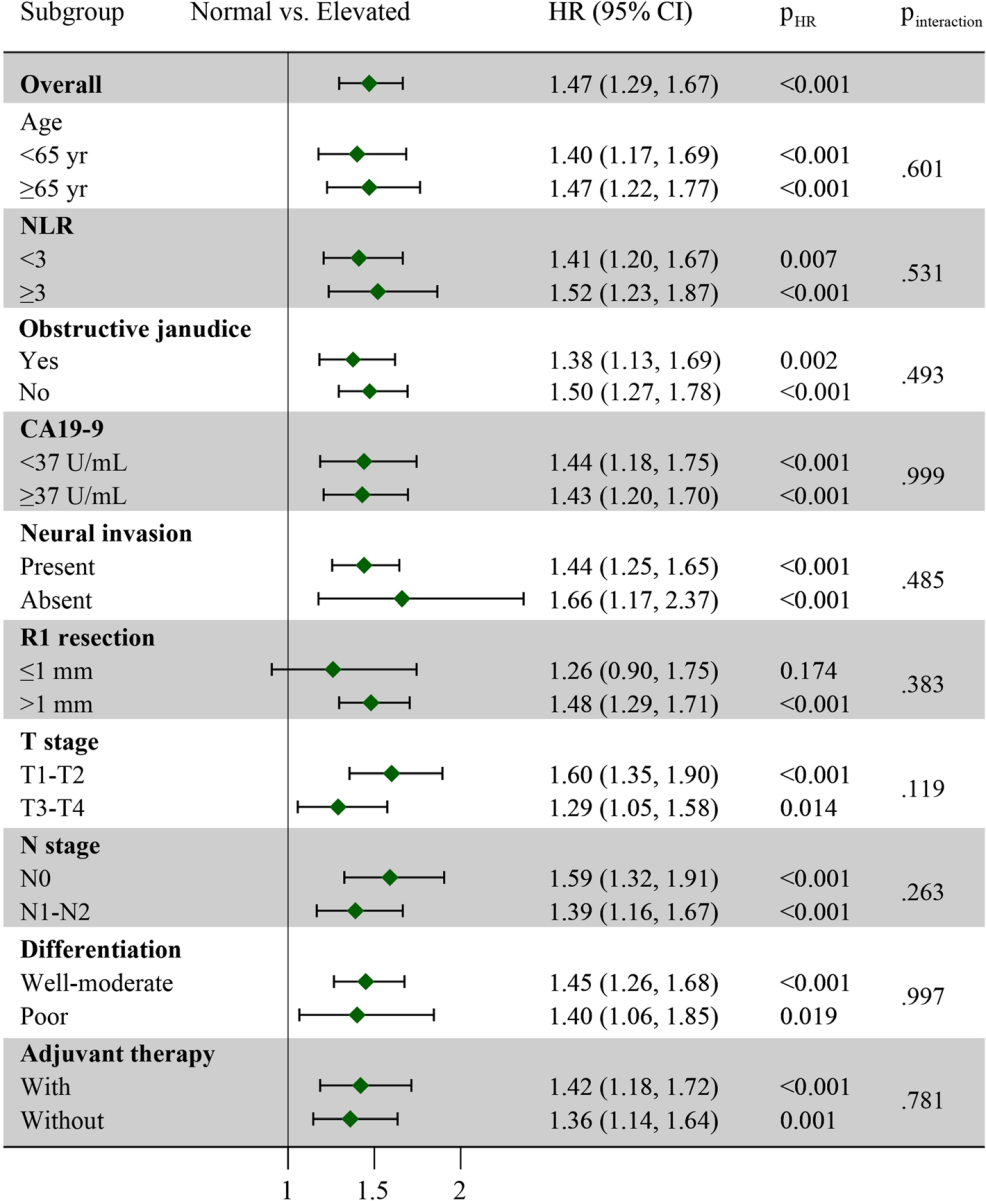
Relationship between the D-dimer and overall survival. HR plots of overall survival rate according to the D-dimer levels are shown for the total cohort. HR, hazard ratio; CI, confidence interval; NLR, neutrophil lymphocyte ratio; CA19-9, serum carbohydrate antigen 19-9

To further investigate whether D-dimer remained a prognostic factor in certain patient subgroups, subgroups analysis was then performed (Fig. 3). Forest plot demonstrated that D-dimer was an independent reliable indicator for OS. However, no significant difference in OS was found in patients with R1 resection (p = 0.174).

### Combination of D-dimer and other preoperative biomarkers enhances prognostic accuracy before operation

Multivariate analysis revealed preoperative parameters, including D-dimer, CA19-9, and NLR, were independent prognostic factors for overall survival in resected PDAC patients. We therefore compared the prognostic value of these parameters and combined them to generate a more accurate prognostic model (Table 3). The C-index of the combination of CA19-9 and NLR was 0.557 and the corresponding AIC value was 13322.06. When combining all three preoperative parameters, the C-index was 0.582, and the corresponding AIC values were 13293.50. Thus, combination of D-dimer, CA19-9, and NLR can enhance the prognostic accuracy for OS before operation.

**Table 3:** Prognostic value of preoperative parameters for PDAC patients receiving radical surgery

	Concordance index		AIC	Likelihood ratio Х^2^
	C-index	Bootstrap 95% CI		
D-dimer	0.551	0.537-0.565	13322.60	34.26
CA19-9	0.543	0.529-0.558	13328.66	28.21
NLR	0.528	0.514-0.542	13343.06	13.81
CA19-9+ NLR	0.557	0.541-0.574	13322.06	36.80
D-dimer+ CA19-9	0.575	0.559-0.591	13300.78	58.50
D-dimer+ CA19-9+ NLR	0.582	0.566-0.598	13293.50	67.36

*PDAC*, pancreatic ductal adenocarcinoma; *AIC*, Akaike information criterion; *C-index*, concordance index; *CI*, confidence interval; *CA19-9*, carbohydrate antigen 19-9; *NLR*, neutrophil lymphocyte ratio

## Discussion

The prognosis of pancreatic cancer patients stays dismal regardless of the rapid progression of surgical techniques and adjuvant therapies. Increasing evidence suggests that not only the intrinsic histopathologic features of the tumor but also the host-related factors are associated with long-term disease outcomes [23–25]. According to new clinical guidelines, considering the special biological behavior of pancreatic cancer, plasma biomarkers and conditional host-related factors are playing more important roles in the decision-making process [23]. In malignancy, together with the host cell inflammatory, cancer cell-specific prothrombotic properties induce the hypercoagulation state [26]. As a consequence, a subclinical activation of blood coagulation is prevalent in cancer patients, as demonstrated by abnormalities of coagulation biomarkers. As a stable fibrin degradation product, plasma D-dimer is a marker of hypercoagulation and is usually used for the assessment of suspected thrombosis or disseminated intravascular coagulation (DIC) in clinical practice [15]. In the present study, preoperative plasma D-dimer was elevated in 30.9% of resectable PDAC patients, and the elevated levels were found to be significantly related to poor prognosis in patients following radical pancreatic surgery.

The D-dimer showed a reliable prognostic value in pancreatic cancer, regardless of age, NLR levels, obstructive jaundice, CA19-9 levels, pathologic staging, poor differentiation, and adjuvant therapy. However, D-dimer is not an accurate prognostic indicator for patients with R1 resection. Several possible reasons might account for it. First, patients with R1 resection are more likely to develop local recurrence when compared to patients with R0 resection, which would negatively affect the prognosis independently [27]. Second, there are only 13.18% of patient with R1 resection, and no statistically significant association was found between D-dimer and R1 resection (p = 0.383). More patients are needed to improve the statistical power.

Previous studies also demonstrated the utility of D-dimer as a prognostic marker in pancreatic cancer. It has been reported that patients with high levels of preoperative plasma D-dimer are at high risk for locally advanced disease or occult hepatic metastases [18]. Subsequent studies have also reported that plasma D-dimer serves as a negative prognostic factor in pancreatic cancer, but the clinical significance remains controversial because of the small number of patients analyzed and the limited perioperative factors included in these studies [19, 28–30]. In addition, Cao et al. [19] examined the prognostic value of preoperative plasma D-dimer in operable pancreatic cancer patients, but the results in this study were not adjusted by other risk factors, and the scale of the study was limited. In a Japanese cohort, Watanabe et al. [20] found that the D-dimer was the only independent prognostic factor in resectable pancreatic cancer. Furthermore, Durczynski et al. [31] found that the D-dimer levels in portal blood were related with poorer overall survival in pancreatic cancer.

However, the mechanism underlying the association between the plasma D-dimer and the prognosis of pancreatic cancer remains unclear. Recent studies have demonstrated the bidirectional association between coagulation and cancer, and cancer-related hypercoagulation is reported to be closely related to cancer progression [12, 32]. Multiple and interdependent processes between the tumor and the patient induce a hypercoagulable state, including tumor-procoagulant activity, host inflammatory responses, and cancer treatments [12]. Tissue factor (TF) is the main initiator of the coagulation cascade, which is highly expressed due to cancer [33]. In addition, in malignancy, TF is also overexpressed by host normal blood cells triggered by cancer-derived inflammatory stimuli [26]. Our studies also found that D-dimer correlated with NLR, an inflammatory indicator, suggesting the relationship between host inflammatory responses and cancer-related hypercoagulation.

On the other hand, some evidence suggested that hypercoagulable state can contribute to cancer progression in turn [32]. TF expression in cancer is related with a variety of pathologic processes, such as thrombosis, metastasis, tumor angiogenesis, and tumor growth [34]. The mesh of fibrin, induced by TF surface expression, was found to envelop cancer cells preventing them from being recognized by NK cells. Furthermore, the formation of platelet-fibrin rich microemboli could help tumor cells escape NK-mediated immune recognition [35]. In vitro experiments, inhibition of TF, FXa, or thrombin has been shown to prevent the formation of metastasis in melanoma tumors [36]. Besides that, a growing body of evidence has suggested that anticoagulants have antitumor effects and can increase the survival time in solid tumor patients [37–40]. Klerk et al. reported that combining low-molecular-weight heparin (LMWH) with other adjuvant therapies improved prognosis in patients with advanced malignancy [38]. In advanced pancreatic cancer patients, the addition of LMWH to gemcitabine-based chemotherapy significantly improved the response and survival [41]. These findings regarding coagulation and cancer supported a pathological role for procoagulant activity in cancer. However, further studies associated with the antitumor effects of anticoagulants are needed in the operable pancreatic cancer patients, especially in patients with elevated D-dimer levels.

Some limitations in this study also warrant emphasis. First, because this was a retrospective study, the use of postoperative prophylactic anticoagulant therapy was not available in the database. Second, patients with neoadjuvant therapy or preoperative anticoagulation treatment were excluded, which may limit the generalizability of this study. Third, the kinetic of serum D-dimer were not analyzed in this study. Further studies are needed to identify the prognostic implications of the kinetic of D-dimer [42].

## Conclusions

Here, we presented one of the largest studies on the prognostic value of different preoperative biomarkers, and found that preoperative plasma D-dimer is an independent indicator for PDAC patients after radical surgery. D-dimer appears to be a promising and reliable indicator for improving the prognostication of patients with pancreatic cancer before surgery. Combination of D-dimer, CA19-9, and NLR can further improve the prognostic accuracy before operation for PDAC patients. These findings also emphasize the importance of coagulation biomarkers and the role of the coagulation system in pancreatic cancer.

## Supplementary Information

**Additional file 1: Supplementary Table 1**. Baseline characteristics.

**Additional file 2: Supplementary Table 2**. Multivariate Cox proportional-hazard regression analysis for overall survival in the open surgery group and robotic surgery group.

## Data Availability

The datasets used and analyzed during the present study are available from the corresponding author on reasonable request.

## Abbreviations

PDAC: Pancreatic ductal adenocarcinoma
OS: Overall survival
DVT: Deep venous thrombosis
PE: Pulmonary embolism
DIC: Disseminated intravascular coagulation
BMI: Body mass index
NLR: Neutrophil lymphocyte ratio
CA19-9: Carbohydrate antigen 19-9
AJCC: American Joint Committee on Cancer
C-index: Concordance index
AIC: Akaike information criterion
CI: Confidence interval
