# Supplementary material for: Preoperative plasma D-dimer independently predicts survival in patients with pancreatic ductal adenocarcinoma undergoing radical resection
Source: World J Surg Oncol. 2021 Jun 9;19:166. doi: 10.1186/s12957-021-02281-8 (PMC8191214; doi:10.1186/s12957-021-02281-8)
Supplement: Supplementary file 1 — Additional file 1: Supplementary Table 1. Baseline characteristics. [file 12957_2021_2281_MOESM1_ESM.docx]

Supplementary Table 1. Baseline characteristics.

| Patient characteristics | Total cohort (n=1351) |
| --- | --- |
| Age | 63 (58-69) |
| Sex (male) | 848 (62.77%) |
| BMI, kg/m^2^ | 22.7 (20.7-24.5) |
| Platelets, ×10^9^ | 187 (150-231) |
| Albumin, g/L | 39 (36-42) |
| NLR | 2.50 (1.92-3.45) |
| CA19-9, U/mL | 147.9 (41.0-443.7) |
| Preoperative biliary drainage | 245 (18.13%) |
| Tumor location |  |
| Head | 857 (63.43%) |
| Body/tail | 494 (36.57%) |
| Surgical approach |  |
| Open | 1039 (76.91%) |
| Robotic | 312 (23.09%) |
| Vessel reconstruction | 191 (14.14%) |
| Postoperative DVT | 3 (0.22%) |
| Postoperative haemorrhage | 53 (3.92%) |
| Nerve plexus invasion | 1155 (85.49%) |
| R1 resection, ≤1 mm | 114 (12.18%) |
| T stage |  |
| T1 | 223 (16.51%) |
| T2 | 620 (45.89%) |
| T3 | 186 (13.77%) |
| T4 | 322 (23.83%) |
| N stage |  |
| N0 | 705 (52.18%) |
| N1 | 502 (37.16%) |
| N2 | 144 (10.66%) |
| TMN stage |  |
| I | 475 (35.16%) |
| II | 459 (33.97%) |
| III | 417 (30.87%) |
| Tumor differentiation |  |
| Well | 498 (36.86%) |
| Moderate | 615 (45.52%) |
| Poor | 238 (17.62%). |
| Adjuvant therapy | 766 (56.70%) |

BMI, body mass index; NLR, neutrophil lymphocyte ratio; CA19-9, serum carbohydrate antigen 19-9; DVT, deep vein thrombosis.

p value < 0.05 indicates statistical significance (in bold)
