# Supplementary material for: Preoperative plasma D-dimer independently predicts survival in patients with pancreatic ductal adenocarcinoma undergoing radical resection
Source: World J Surg Oncol. 2021 Jun 9;19:166. doi: 10.1186/s12957-021-02281-8 (PMC8191214; doi:10.1186/s12957-021-02281-8)
Supplement: Supplementary file 2 — Additional file 2: Supplementary Table 2. Multivariate Cox proportional-hazard regression analysis for overall survival in the open surgery group and robotic surgery group. [file 12957_2021_2281_MOESM2_ESM.docx]

Supplementary Table 2. Multivariate Cox proportional-hazard regression analysis for overall survival in the open surgery group and robotic surgery group

|  | Open surgery (n=1039) | |  | Robotic surgery (n=312) | |
| --- | --- | --- | --- | --- | --- |
|  | **Hazard ratio** | **p** |  | **Hazard ratio** | **p** |
| Age, ≥65 yr | 1.18 (1.02-1.37) | **0.023** |  | 1.11 (0.82-1.49) | 0.509 |
| Sex (male) | 1.10 (0.95-1.27) | 0.226 |  | 1.06 (0.78-1.43) | 0.702 |
| Platelets, ≥200×10^9^ | 1.14 (0.98-1.32) | 0.089 |  | 0.79 (0.58-1.07) | 0.134 |
| NLR, ≥3 | 1.10 (0.95-1.28) | 0.185 |  | 1.26 (0.93-1.71) | 0.134 |
| Albumin, <40 g/L | 1.13 (0.97-1.30) | **0.113** |  | 0.91 (0.67-1.24) | 0.559 |
| D-dimer, ≥0.55 ng/mL | 1.21 (1.04-1.40) | **0.012** |  | 1.77 (1.29-2.44) | **<0.001** |
| CA19-9, ≥37 U/mL | 1.30 (1.14-1.51) | **0.001** |  | 0.94 (0.70-1.25) | 0.653 |
| Tumor location |  |  |  |  |  |
| Head | Ref. |  |  | Ref. |  |
| Body/tail | 1.10 (0.94-1.28) | 0.257 |  | 1.05 (0.77-1.42) | 0.775 |
| Major vessel resection | 1.02 (0.82-1.27) | 0.830 |  | 1.78 (1.04-3.06) | **0.037** |
| Neural invasion | 1.33 (1.08-1.63) | **0.007** |  | 1.85 (1.19-2.88) | **0.007** |
| R1 resection, ≤1 mm | 1.36 (1.13-1.64) | **0.001** |  | 1.45 (0.89-2.38) | 0.138 |
| T stage |  |  |  |  |  |
| T1-T2 | Ref. |  |  | Ref. |  |
| T3-T4 | 1.59 (1.34-1.88) | **<0.001** |  | 1.37 (0.96-1.94) | 0.080 |
| N stage |  |  |  |  |  |
| N0 | Ref. |  |  | Ref. |  |
| N1-N2 | 1.47 (1.27-1.70) | **<0.001** |  | 1.58 (1.17-2.14) | **0.003** |
| Differentiation |  |  |  |  |  |
| Well-moderate | Ref |  |  | Ref. |  |
| Poor | 1.60 (1.34-1.91) | **<0.001** |  | 2.54 (1.77-3.65) | **<0.001** |
| Adjuvant therapy | 0.63 (0.54-0.73) | **<0.001** |  | 0.47 (0.35-0.65) | **<0.001** |

NLR, neutrophil lymphocyte ratio; CA19-9, serum carbohydrate antigen 19-9; PDAC, pancreatic ductal adenocarcinoma.

p value < 0.05 indicates statistical significance (in bold)
